# Activation of natural killer cells by rituximab in granulomatosis with polyangiitis

**DOI:** 10.1186/s13075-019-2054-0

**Published:** 2019-12-11

**Authors:** Doris Urlaub, Shuyang Zhao, Norbert Blank, Raoul Bergner, Maren Claus, Theresa Tretter, Hanns-Martin Lorenz, Carsten Watzl, Wolfgang Merkt

**Affiliations:** 1 Leibniz Research Centre for Working Environment and Human Factors at TU Dortmund (IfADo), Dortmund, Germany; 2 Department of Hematology, Oncology and Rheumatology, Internal Medicine V, University Hospital of Heidelberg, Heidelberg, Germany; 3 Department of Rheumatology, Nephrology, Oncology, Klinikum Ludwigshafen, Ludwigshafen, Germany

**Keywords:** Natural killer cells, Rituximab, B cell depletion, Rheumatic diseases, ANCA-associated vasculitis, Granulomatosis with polyangiitis, Obinutuzumab, Antibody-dependent cellular cytotoxicity (ADCC), Fc-γ-receptor IIIa (FcγRIIIa; CD16)

## Abstract

**Objective:**

In the last few years, anti-CD20 antibody rituximab profoundly changed the therapeutic landscape of granulomatosis with polyangiitis (GPA). Here, we investigated whether natural killer (NK) cells may play a role in rituximab’s mechanism of action in GPA.

**Methods:**

B cell depletion, NK cell degranulation, and the expression of CD69 and CD16 on NK cells were measured in a series of in vitro experiments using peripheral blood mononuclear cells (PBMCs). In vivo activation of NK cells was investigated in patients receiving rituximab infusions. Cells were analyzed by seven-color flow cytometry.

**Results:**

NK cells from GPA patients were activated by immobilized rituximab. Also soluble rituximab activated NK cells, provided that B cells were present. NK cells degranulated and expressed the activation marker CD69 while CD16 expression was decreased. This activation of NK cells by soluble rituximab was accompanied by a reduction of B cells. The next-generation anti-CD20 antibody obinutuzumab showed stronger effects compared to rituximab on both the reduction of B cells and the activation of NK cells. Finally, we found that rituximab led to the activation of NK cells in vivo, provided that B cells were not depleted due to prior rituximab infusions.

**Conclusion:**

B cell-bound rituximab activates NK cells in GPA. While NK cells therefore participate in rituximab’s mechanism of action in humans, their potential may be more efficiently exploited, e.g., by Fc engineering of therapeutic antibodies.

## Introduction

Granulomatosis with polyangiitis (GPA) is a non-malignant, life-threatening systemic inflammatory disease [1]. Recently, rituximab has been licensed for both induction and maintenance therapy [2–5], thus profoundly changing the therapeutic landscape of GPA. Despite this important progress, the response to rituximab treatment is not ideal. Only little more than half of the patients achieved complete remission after induction therapy with rituximab [4], and about 40% of patients had minor or major relapses despite rituximab maintenance therapy after 5 years [5]. Biomarkers predicting a good response do not exist, and further questions regarding rituximab treatment in GPA and other inflammatory diseases remain [6, 7]. As a fundamental step to clarify these obscurities, a deeper understanding of rituximab’s mechanism of action in GPA is needed.

Rituximab is a chimeric type I anti-CD20 monoclonal antibody [8]. Its application leads to the depletion of CD20-positive B lymphocytes. Theoretically, direct cytotoxic effects, complement-dependent cytotoxicity (CDC), antibody-dependent cellular cytotoxicity (ADCC) by natural killer (NK) cells, and antibody-dependent cell phagocytosis (ADCP) by monocytes or macrophages may play a role.

The low affinity Fc-γ-receptor IIIa (FcγRIIIa; CD16) plays an important role in rituximab’s mechanism of action [9]. For efficient CD16 stimulation, it is necessary that Fc fragments are organized either on membranes or in immune complexes. CD16 is expressed on myeloid and NK cells. The importance of CD16 for rituximab’s efficacy in humans is sustained by numerous clinical associations in lymphoma studies [10–14]. The high-affinity single nucleotide polymorphism (SNP) of the CD16 gene (158 V) is associated with a good clinical response to rituximab in rheumatoid arthritis and systemic lupus [15–17], pointing towards a role of CD16 also in the treatment of systemic inflammatory diseases. Together, these studies establish CD16 as an important determinant of rituximab’s efficacy.

On NK cells, CD16 is the only Fc receptor and possesses a prominent activating function [18]. An involvement of NK cells in rituximab treatment is therefore intuitive, but less well investigated. Studies in vivo in humans [14], in vitro [19, 20], and in a humanized mouse model [21] indicate that NK cells contribute to cancer therapy with rituximab.

In contrast, CD16 and NK cells have hardly been studied in GPA. The abovementioned correlation between high-affinity SNP 158 V and response to rituximab was not found in GPA [22]. CD16 expression was diminished on NK cells from GPA patients as compared to healthy individuals [23].

**Fig. 1:**
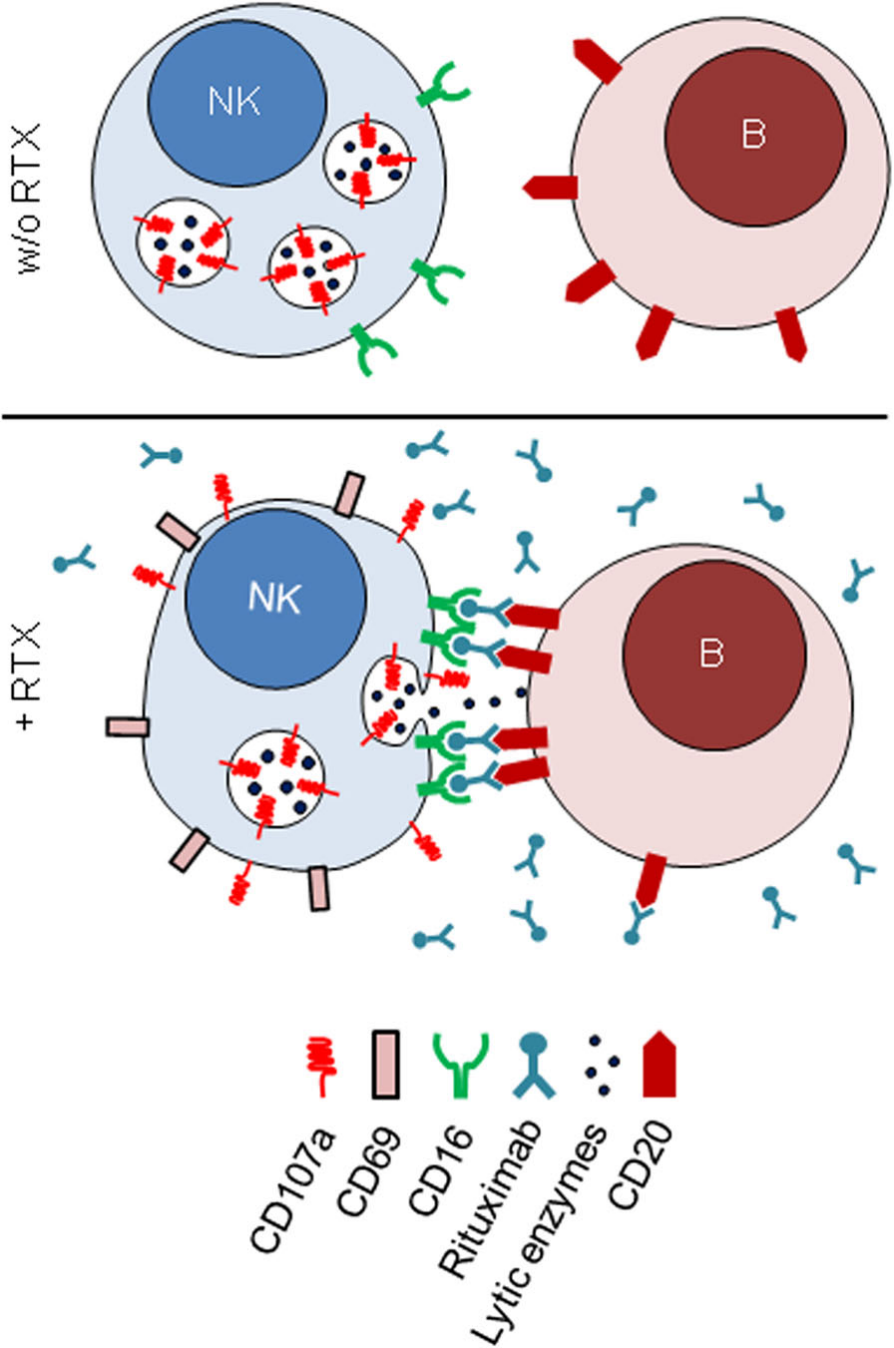
Schematic overview of NK cell-mediated antibody-dependent cellular cytotoxicity (ADCC) induced by rituximab, as described previously in a non-malignant experimental setting [24]

Beyond that, it is still unclear whether NK cells can lyse rituximab-coated non-malignant B cells despite its broad application in inflammatory diseases like GPA. In non-malignant conditions, B cells do not express tumor-associated NK cell receptor ligands [20]. In addition, immune disturbances in inflammatory diseases may impact the functionality of rituximab. We therefore investigated in a preceding study whether NK cells can lyse autologous non-malignant (healthy) B cells in the presence of rituximab, which was the case [24] (see schematic overview, Fig. 1).

To date, NK cells have not been investigated in the context of rituximab in GPA. Here, we show that CD16 on GPA NK cells is functional. GPA NK cells recognize rituximab and degranulate in the presence of rituximab-bound B cells. Accordingly, NK cells are activated by rituximab in GPA patients in vivo. We further show that obinutuzumab, a type 2 anti-CD20 antibody with similar affinity to CD20 but enhanced affinity to CD16 [25] is more potent in activating GPA NK cells than rituximab.

## Materials and methods

### Donor consent and ethical approval

The ethics committee of the University of Heidelberg approved this study. Written informed consent was obtained from healthy donors and patients. In total, 47 blood withdrawals took place (pre- and post rituximab withdrawals for determination of in vivo effects on NK cells were counted as only one withdrawal). All patients suffered from GPA, except two patients included in the in vivo cohort who suffered from rheumatoid arthritis and eosinophilic granulomatosis with polyangiitis, respectively. The numbers of patients included in individual experiments are disclosed in the figure legends. Individual patients were included only once per experiment and analysis.

### PBMC preparation

Peripheral blood mononuclear cells (PBMCs) were isolated from heparinized blood from healthy volunteers and patients by density gradient centrifugation using Biocoll separating solution (Biochrom Ltd., Cambourne, UK). PBMCs were frozen in fetal calf serum (FCS) with 10% dimethyl sulfoxide. PBMCs were thawed and transferred in culture medium (RPMI 1640 supplemented with 10% FCS, 1% L-glutamine, 15 mM HEPES buffer, and 1% penicillin/streptomycin). After resting overnight [26], PBMCs were used for experiments.

### Stimulation of PBMCs on plates coated with monoclonal antibodies

Ninety-six flat bottom well plates were coated by incubation overnight on 4 °C with phosphate-buffered saline (PBS) containing 5 μg/ml of antibodies. Plates were washed with PBS before PBMCs were added.

### Stimulation with therapeutic antibodies

PBMCs were cultured overnight with 10 μg therapeutic antibody per milliliter of culture medium. Alternatively, plates were coated as described above. Anti-CD107a PE-Cy5 (BD Biosciences, San Jose, CA, USA) was added at the beginning of the stimulation. The next morning, PBMCs were washed with PBS, incubated in PBS supplemented with Zombie Aqua™ Fixable Viability Kit (final conc. 1/500; BioLegend, San Diego, CA, USA) and incubated for 20 min on ice. Subsequently, PBMCs were washed in 2% FCS in PBS and incubated for 30 min on 4 °C in 2% FCS in PBS supplemented with anti-CD56 Brilliant Violet 421 (Clone NCAM16.2; BD), anti-CD16 FITC (Immunotech, Marseille, FRA), anti-CD3 APC (UCHT1), anti-CD19 APC-Cy7 (HIB19), and anti-CD69 PE (all from BioLegend). After a final washing step, PBMCs were directly analyzed on a three-laser flow cytometer (LSRII, BD). Data were processed using FlowJo® software (FlowJo LCC, Ashland, OR, USA). The gating strategy is shown in Additional file 1.

### Alternative PBMC preparation and stimulation with antibodies (Fig. 4a–e)

PBMCs from healthy donors were purified by density gradient centrifugation over lymphocyte separating medium (PAN Biotech). PBMCs were frozen in order to allow comparison with GPA patient probes that were transported deep-frozen to our collaboration partner. Thawed PBMCs from patients or healthy donors were incubated overnight in medium (IMDM with Glutamax™, 10% FCS, 1% Penicillin/Streptomycin, all from Gibco) with a final concentration of 10 μg/ml rituximab, infliximab, or obinutuzumab and without antibody as control. Next day, cells were washed and stained first with Zombie Yellow (BioLegend) in PBS for 15 min at room temperature, then stained with anti-CD56 Brilliant Violet 421 (NCAM16.2, BD), anti-CD3 FITC (Hit3a, BioLegend), anti-CD19 AlexaFluor700 (Hib19, BioLegend) or anti-CD19 AlexaFluor647 (SJ25-C1, Life Technologies), and anti-CD16 PE (3G8, BioLegend).

### Measurement of in vivo NK cell activation

Blood was withdrawn before the start—in the morning of the same day—and directly after the end of RTX infusions, which were performed as usual in clinical routine. PBMCs were isolated by density gradient centrifugation and frozen. For analysis, PBMCs were thawed, washed with PBS, and incubated for 20 min on ice in dark in PBS supplemented with Zombie Aqua™ fixable viability dye, according to the manufacturer’s recommendations (final concentration 1/500; BioLegend). PBMCs were then washed and incubated for 30 min in a 4 °C fridge in PBS supplemented with 2% fetal calf serum and the following cocktail of monoclonal antibodies: anti-CD56 Brilliant violet 421 (Clone NCAM16.2, BD Biosciences, San Jose, CA, USA), anti-CD3 APC-Cy7 (UCHT1), anti-CD19 APC (HIB19), and anti-CD69 PE (FN50) (all from BioLegend). Finally, PBMCs were washed, resuspended in 2% FCS in PBS containing 2% paraformaldehyde, and analyzed on a three-laser cytometer (LSRII, BD Biosciences). Data were processed using FlowJo® software (FlowJo LCC). The gating strategy is shown in Additional file 3.

### Statistical analysis

The statistical analysis was performed in an exploratory way using GraphPad’s Prism versions 5 and 8. *p* values have to be interpreted descriptively. The respective tests are given in graph legends or in the text and were performed with a significance level of 5%. In graphs, *p* values of < 0.05, < 0.01, and < 0.001 are represented by stars (*, **, and ***, respectively).

## Results

### GPA NK cells possessed a functional Fc receptor CD16 and recognized immobilized rituximab

**Fig. 2:**
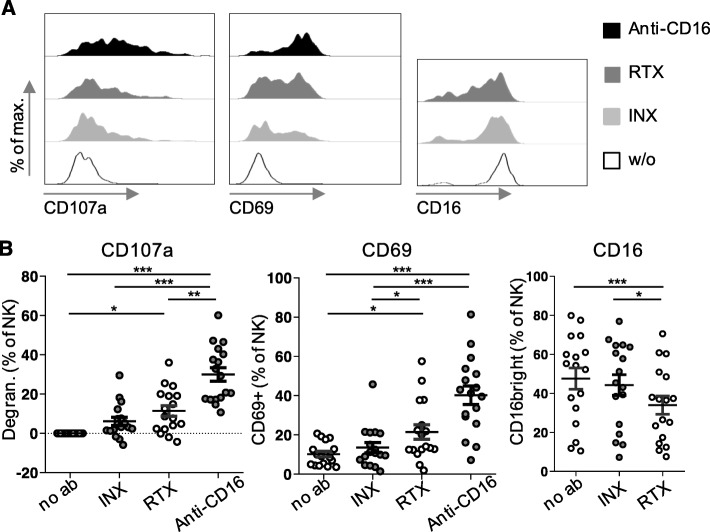
Fc receptor CD16 was functional on GPA NK cells, which also recognized immobilized rituximab. PBMCs from 17 GPA patients were cultured overnight on uncoated plates (*w/o* or *no ab*) or on plates coated with rituximab (RTX), infliximab (INX), or anti-CD16 in the presence of a fluorochrome-labeled anti-CD107a antibody. Thereafter, PBMCs were stained with a viability dye and fluorochrome-labeled antibodies against CD3, CD19, CD56, CD16, and CD69 and analyzed by flow cytometry. The gating strategy is shown in Additional file 1. NK cells were defined as viable CD3-CD19-CD56+ lymphocytes. **a** Exemplary histograms show the cumulative surface expression of CD107a during the incubation period and the surface expression of CD69 and CD16 at the end of the incubation period on NK cells originating from the same patient. **b** Summarizing dot plots of all patients. Degranulation was defined as [(% of CD107a+ NK cells after culture with therapeutic antibody) − (% of CD107a+ NK cells after culture without antibody)]. Friedman test confirmed significance (*p* < 0.0001, *p* < 0.0001 and *p* = 0.0010, respectively). Significant Dunn’s post tests are indicated by stars

A previous study revealed a decreased expression of CD16 on NK cells in patients with GPA [23]. Therefore, we first examined whether CD16 was functional on NK cells from GPA patients: PBMCs were cultured overnight on plates coated with an anti-CD16 antibody (clone 3G8), which has a high affinity for CD16 and is known to activate NK cells. This induced degranulation and upregulation of the activation marker CD69 (Fig. 2).

To investigate if CD16 could be stimulated by its natural ligands, we also cultured PBMCs overnight on plates coated with rituximab or infliximab. The Fc parts of both antibodies are potential low-affinity ligands for CD16. We used the tumor necrosis factor alpha (TNFα) inhibitor infliximab as a non-B-cell-depleting control. Similarly to rituximab, Infliximab is an intravenously applied IgG1 therapeutic antibody used to treat rheumatic diseases. We assessed three different surrogates of NK cell activation, i.e., degranulation, upregulation of CD69, and downregulation of CD16. Degranulation measured by CD107a upregulation can also be regarded as a correlate of cytotoxicity [27]. All three surrogates indicated that immobilized rituximab induced NK cell activation. Immobilized infliximab showed a tendency to activate NK cells, but without reaching statistical significance (Fig. 2).

These data show that CD16 was in principle functional on GPA NK cells and that these cells could recognize rituximab.

### B cells from GPA patients were reduced upon in vitro incubation of PBMCs with rituximab in the absence of serum factors

Within PBMCs from healthy donors, rituximab-induced B cell depletion takes place in the absence of complement factors (i.e., in medium with heat-inactivated FCS) and is dependent on the amount of NK cells [24]. We tested whether rituximab-induced B cell depletion takes place also in PBMCs from 15 GPA patients. In this explorative cohort, PBMCs were cultured with or without soluble rituximab overnight in conical centrifuge tubes. B cells were significantly reduced upon in vitro incubation with rituximab (*p* < 0.01 using Wilcoxon test; data not shown).

### Rituximab induced NK cell degranulation and activation depending on the presence of B cells

**Fig. 3:**
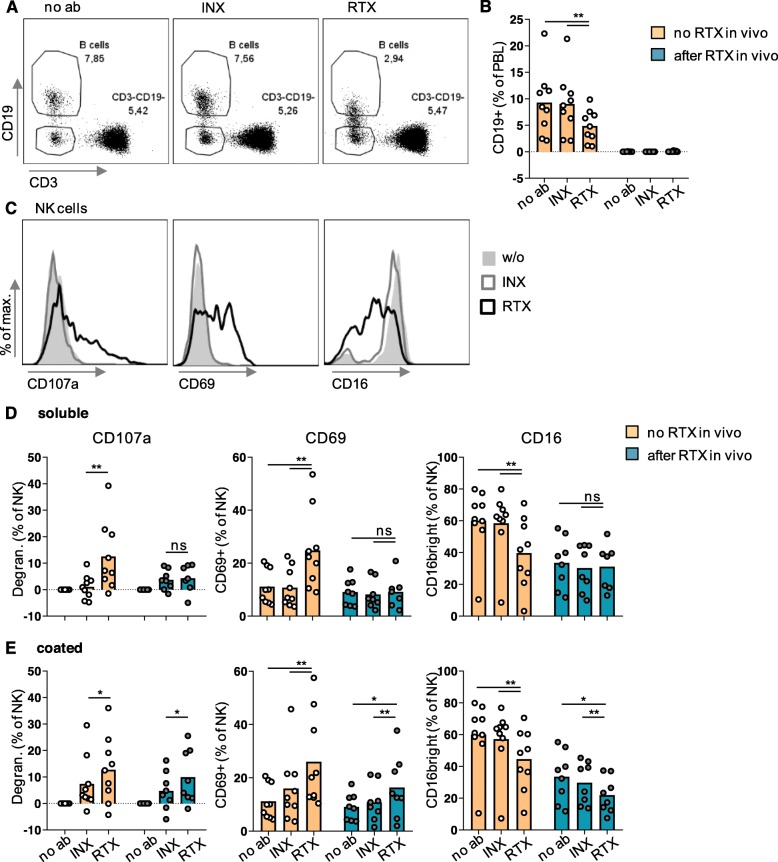
Simultaneously to B cell reduction, Rituximab led to degranulation and activation of GPA NK cells—provided that rituximab was bound to a surface. PBMCs from the 17 GPA patients shown in Fig. 2 were divided into two groups: nine were rituximab-naive and eight rituximab-experienced. PBMCs were cultured overnight with or without soluble or coated rituximab (RTX), infliximab (INX), or anti-CD16 antibody in the presence of a fluorochrome-labeled anti-CD107a antibody. PBMCs were then stained and analyzed as described in Fig. 2. **a, b** B cells were defined as life CD3-CD19+ lymphocytes (PBL). **a** Exemplary dot plots originating from the same rituximab-naive patient shown in Fig. 2a. As a fraction of B cells seemed to lose CD19 upon incubation with rituximab and as the viability dye cannot exclude early apoptotic cells, we provide data in Additional file 2 showing that the loss of CD19 is associated with cell death. **b** Percentages of B cells as a function of in vivo and in vitro treatment with rituximab. **c**-**e** NK cells were defined as viable CD3-CD19-CD56+ PBL. **c** Exemplary histograms originating from PBMCs from the patient after incubation with soluble antibodies; w/o, without antibody. **d-e** Degranulation was defined as [(% of CD107a+ NK cells after culture with therapeutic antibody) − (% of CD107a+ NK cells after culture without antibody)]. Degranulation, CD69 expression, and percentage of CD16bright cells were analyzed. Statistical analyses for comparison of groups of biological interest were performed using Wilcoxon test and are indicated in the graphs. Bars represent means

We next wanted to know whether in vitro depletion of GPA B cells was associated with NK cell activation. In a series of experiments, PBMCs were cultured on flat-bottom plates under varying conditions (Fig. 3). Soluble infliximab was included as non-B-cell-depleting IgG1 control. B cells from patients that had received rituximab infusions prior to study inclusion were already depleted (Fig. 3b). We therefore analyzed these patients separately. B cells were reduced upon in vitro incubation with soluble rituximab (Fig. 3a,b). Stimulation with soluble anti-CD16 antibody was performed in seven of the nine rituximab naive patients and did not lead to B cell depletion (not shown).

After overnight incubation, markers of NK cell activation were analyzed. In rituximab-naive patients, NK cells degranulated significantly stronger in the presence of soluble rituximab as compared to infliximab (Fig. 3c,d). In contrast, in rituximab-experienced patients lacking B cells, there was no difference in degranulation between treatment with soluble rituximab and infliximab in vitro. However, NK cell activation was restored when antibodies had been immobilized by coating to the plate (Fig. 3e). This restoration by coating indicates that NK cells in rituximab-experienced patients were functional. The missing degranulation was rather due to missing B cells or, to be more precise, missing surface binding of rituximab. These findings were confirmed using CD69 upregulation and CD16 downregulation as alternative surrogates of activation (Fig. 3c–e). Together, rituximab activated GPA NK cells, provided that it was bound to a (cellular) surface.

Additionally, we observed that the percentage of CD16bright cells among NK cells was significantly lower in rituximab-experienced than rituximab-naive patients in untreated PBMCs (“no ab”) (*p* = 0.0079, Wilcoxon test) (Fig. 3d, right panel). Nevertheless, in vitro culture on rituximab-coated plates was associated with a further decrease of CD16 (Fig. 3e, right panel).

**Fig. 4:**
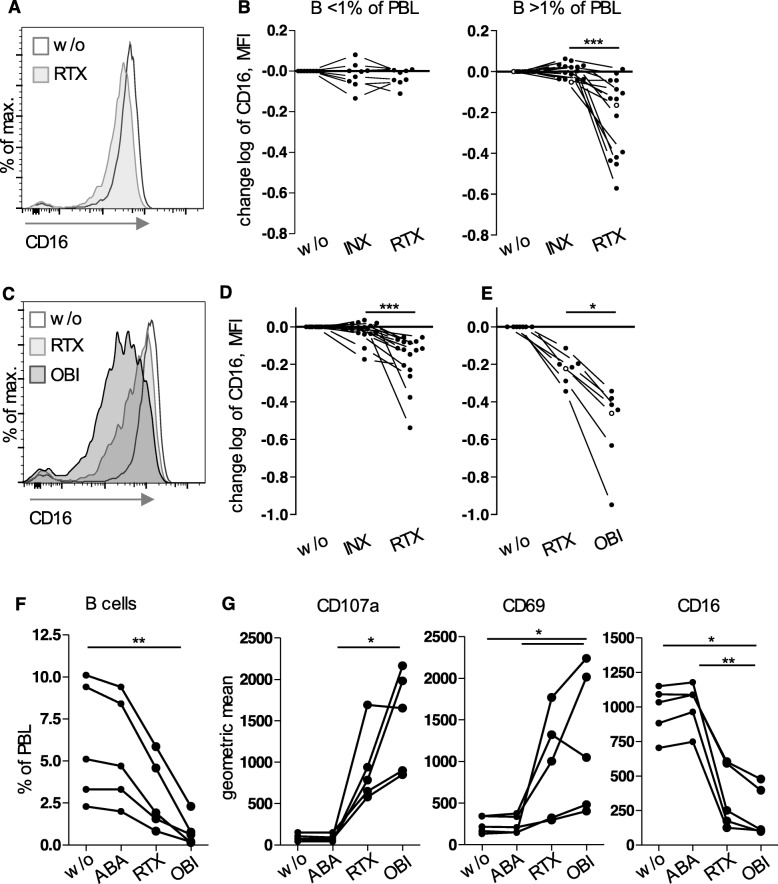
Obinutuzumab induced stronger effects than rituximab. **a, b** PBMCs from GPA patients were treated with soluble antibodies like in Fig. 3. Experiments with PBMCs without relevant amounts of B cells (< 1% of PBL, *n* = 9) are depicted separately. > 1% of PBL, *n* = 15. **a** CD16 expression on NK cells from an example donor; this donor is characterized by an open circle in **b**. **b** As MFI values strongly varied between donors, data were normalized to the sample w/o antibody and logarithmized (change log) in order to simplify comparisons. RTX had a significant effect only in PBMCs with > 1% B cells (Wilcoxon test, *p* < 0.0001). **c–e** Using corresponding methods, PBMCs from healthy donors were cultured with either INX and RTX (*n* = 16, **d**) or RTX and obinutuzumab (OBI) (*n* = 7, **e**). **c** Example CD16 expression; this donor is characterized by an open circle in **e**. **d, e** Wilcoxon tests revealed significant differences (*p* < 0.0001 and *p* = 0.0156, respectively). **f, g** PBMCs from five rituximab-naive GPA patients were cultured overnight with RTX or OBI. In addition, IgG1 containing abatacept (ABA) was used as control. **f** B cell percentages. **g** Degranulation and expression of activation markers on NK cells. Geometric means after gating on NK cells are shown. *p* values determined by Friedman tests for B cells (**f**), CD107a, CD69, and CD16 (**g**) were < 0.0001, = 0.0002, = 0.0006, and < 0.0001 respectively. Significant post tests as indicated

In order to confirm NK cell activation by rituximab under slightly different experimental and analytical conditions, PBMCs from GPA patients were cultured overnight in another laboratory with rituximab, infliximab, or without therapeutic antibody (Fig. 4a,b). In conformity with the results presented above, CD16 was selectively downregulated by soluble rituximab, but only in the presence of B cells (Fig. 4b).

### The next-generation anti-CD20 antibody obinutuzumab induced stronger effects than rituximab

As these results strongly suggested an involvement of NK cells and CD16 in depleting B cells from GPA patients, we hypothesized that obinutuzumab which is characterized by an increased affinity to CD16 would lead to stronger NK cell activation. We first tested this hypothesis by using PBMCs from healthy donors and treating them with soluble infliximab or rituximab, or with soluble rituximab or obinutuzumab. As seen in PBMCs from B cell sufficient GPA patients, rituximab resulted in a significant downregulation of CD16 on NK cells compared to infliximab or the control without therapeutic antibody (Fig. 4d). Interestingly, upon obinutuzumab treatment, we observed an even stronger CD16 downregulation compared to rituximab (Fig. 4c–e). When testing obinutuzumab on PBMCs from GPA patients, we observed more pronounced B cell depletion (Fig. 4f) and NK cell activation as determined by degranulation and CD69 and CD16 expression (Fig. 4g).

This demonstrates that the affinity to CD16 altered the effect of anti-CD20 antibodies on GPA NK cells. These data also showed that rituximab did not exploit the maximum potential of NK cells.

### Rituximab activated NK cells in vivo in patients with systemic inflammatory disease

**Fig. 5:**
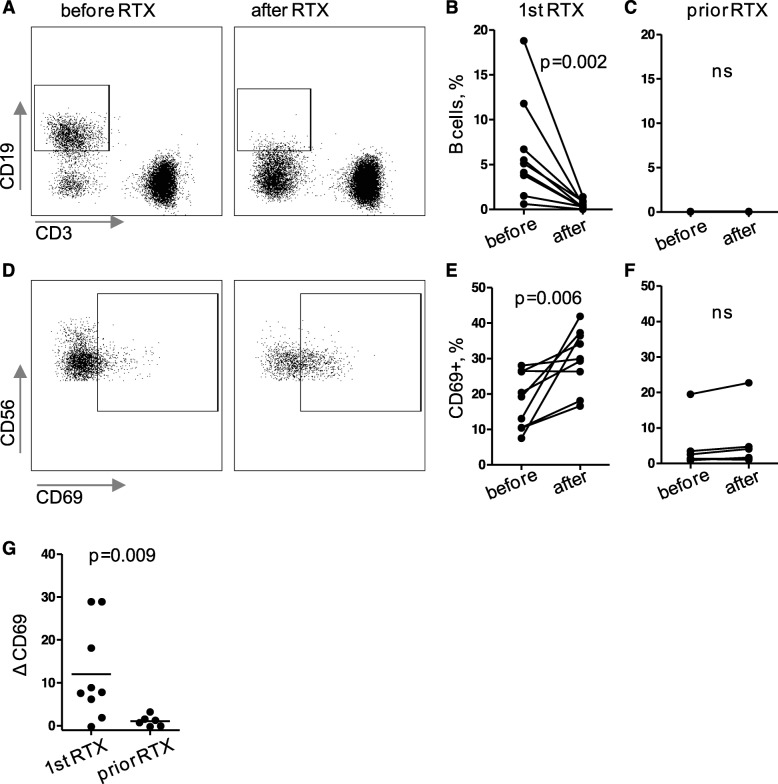
Rituximab activated NK cells in vivo in patients with systemic inflammatory disease. PBMCs were isolated from blood withdrawn before and directly after rituximab (RTX) infusions from 15 patients (13 GPA, 1 eosinophilic GPA, and 1 rheumatoid arthritis). PBMCs were incubated with a panel of fluorochrome-labeled antibodies in order to determine percentages of B cells among lymphocytes (**a-c**) and the activation surrogate marker CD69 on NK cells (**d-g**) by flow cytometry. **a**, **d** Exemplary dot plots, all originating from the same patient receiving RTX for the first time. Statistical analysis of data from patients that received RTX for the first time (*n* = 9, 1st RTX; **b**, **e**) was performed separately from analysis from patients whose B cells were depleted due to prior rituximab infusions (*n* = 6, prior RTX; **c**, **f**), using Wilcoxon test. ns, not significant. **g** Direct comparison of patients receiving RTX for the first time or not, using one-tailed Mann-Whitney test. *N* = 6 per group; ΔCD69, (percentages of CD69+ NK cells after RTX) − (percentages of CD69+ NK cells before RTX); crossbars, means

In order to check for in vivo relevance of these findings, we sampled blood from 15 patients with systemic inflammatory disease before and after rituximab infusions. Thirteen patients suffered from GPA, 1 from eosinophilic GPA, and 1 from rheumatoid arthritis. Nine patients were rituximab-naive (including the patients with eosinophilic GPA and rheumatoid arthritis), and B cells of these patients were significantly reduced upon their first rituximab infusion (Fig. 5a,b). The remaining six patients had received rituximab prior to study inclusion; B cells of these patients were already depleted (Fig. 5c). Therefore, we analyzed rituximab-naive and rituximab-experienced patients separately.

This subgroup analysis showed that CD69 upregulation was only significant in rituximab-naive patients (Fig. 5d–g), even though the CD69 expression was already higher before the infusion, presumably because of disease activity [23]. Within the naive group, we observed some donor-dependent variability with few patients showing low effects of rituximab on NK cell activation. In contrast, in the rituximab-experienced group, NK cells from all patients were hardly activated by rituximab (Fig. 5e,g).

Together, NK cells from patients with systemic inflammatory disease could be activated by rituximab in vivo.

## Discussion

Nowadays, NK cells are well-accepted mediators of cytotoxic antibodies in cancer therapies. Consequently, more targeted “NK cell therapies” are being established in order to better exploit their cytotoxic potential [28, 29]. In contrast to the cancer field, the role of NK cells is still poorly defined in the treatment of systemic inflammatory diseases with the cell-binding therapeutic antibody rituximab.

It was so far unclear whether NK cells are involved in depleting B cells upon treatment with rituximab in GPA. In a preceding study [24], we have shown in a series of NK cell depletion and re-substitution assays that rituximab-bound healthy B cells were depleted depending on the amount of NK cells indicating that NK cells can kill non-malignant rituximab-coated B cells, at least in vitro. Unfortunately, it was not possible in the present study to withdraw sufficient amounts of blood from GPA patients that would allow for similar re-substitution experiments as performed with healthy donors before. Therefore, we have chosen another way to investigate NK cell involvement in rituximab treatment in GPA. In vitro, we observed B cell depletion in parallel to NK cell activation surrogates. All investigated activation surrogates were altered by rituximab, including a marker of degranulation, CD107a. These alterations were similar to those observed in other situations in which NK cells performed rituximab-induced ADCC (see schematic overview, Fig. 1). The finding that this NK cell activation was dependent on the presence of B cells or on immobilization of rituximab to the plastic surface adds some specificity. This relevance of surface binding matches well to the known necessity of clustering of epitopes for full CD16-mediated stimulation (see the “Introduction” section). It cannot be completely ruled out, though, that NK cells were activated indirectly, e.g., by release of cytokines from activated monocytes which may also bind to rituximab. In vivo, NK cells were similarly activated upon rituximab infusion. This was again dependent on the presence of B cells. These data provide important information lacking so far in non-malignant diseases. Together, they establish that NK cells are activated by rituximab in GPA and thus involved in its mechanism of action.

It was unclear before whether blood NK cells from GPA patients were fully functional because they were found to be activated and to possess reduced levels of CD16 in a previous study [23]. The present study, however, showed that engaging CD16 significantly enhanced activation of GPA NK cells, which also recognized immobilized rituximab. Consequently, they could be (further) activated by cell-bound rituximab. In line with this, our in vivo data showed significant add-on activation directly after rituximab infusion despite elevated CD69 levels in rituximab-naive (and therefore likely active) GPA patients. NK cell pre-activation due to disease activity does therefore not rule out their contribution to the effects of rituximab treatment. This evidence was also lacking before.

Despite the involvement of NK cells in rituximab’s mechanisms of action, the quantitative contribution of NK cells in an individual patient may be controversially discussed. In particular, the degree of CD69 upregulation in vivo was variable. Two patients showed almost no CD69 upregulation while B cells were strongly reduced in every patient. We believe that several mechanisms of action take place in parallel, similar to the current view of rituximab in cancer therapy. Multiple factors that can differ between patients potentially determine the response to rituximab. It has also been suggested that CDC and ADCC may negatively interfere with each other [30, 31], while we found a rather synergistic function of serum factors and NK cells in depleting non-malignant human B cells [24].

Our data further indicate that rituximab-induced B cell depletion takes place in the blood and is carried out within a few hours.

Finally, our comparison of rituximab with obinutuzumab showed that Fc engineering can increase NK cell activation and their efficiency to kill non-malignant B cells in vitro. So far, it is unknown if patients with non-malignant diseases would profit from this therapy.

## Conclusion

We conclude that NK cells are activated by rituximab in GPA patients. This activation is sub-maximal. NK cells are cytotoxic cells whose potential may be more efficiently exploited by Fc-engineered therapeutic antibodies.

## Supplementary information

**Additional file 1: Figure S1.** Gating strategy for in vitro experiments. Gating was performed in a standardized way, and an example GPA patient is shown. **a** First, live cells were roughly gated based on forward and sideward scatter (FSC, SSC). Second, Zombie Aqua™ viability dye positive cells were determined as “dead” and remaining cells as “live”; for this purpose dot plots showing CD56-Bv421 and Zombie aqua dye were used in order to confirm correct compensation as these fluorochromes were excited by the same laser. After doublet exclusion, peripheral blood lymphocytes (PBL) were gated in a conservative, “tight” fashion to exclude monocytes and, as good as possible, potentially apoptotic cells which would be located on the upper left part of the main population. Among PBL, T cells were determined as CD3 + CD19−, B cells as CD3-CD19+ and NK cells as CD3-CD19-CD56+ cells. **b** Within the NK cell gate, gates for CD69 positive cells were defined. The same example patient as in a) is shown, after culture with no therapeutic antibody (no ab), with infliximab (INX) or with rituximab (RTX) overnight. Gates for CD107a positive cells and CD16bright cells were defined accordingly.

**Additional file 2: Figure S2.** Loss of CD19 expression was associated with cell death. In order to exclude that reduced numbers of CD19 positive (i.e. CD19 bright) B cells were rather loosing CD19 expression than dying upon incubation with rituximab, PBMCs from healthy donors were incubated without (no ab) or with rituximab (RTX) overnight and subsequently stained with anti-CD3, anti-CD19 and Annexin-V. The gating strategy is shown. The right graphs show overlays of CD3-CD19bright and CD3-CD19dim lymphocytes. Large proportions of CD19dim cells were Annexin-V positive indicating cell death in these cells in both RTX untreated and treated samples. One of three similar experiments is shown. This result was in line with an earlier study [24].

**Additional file 3: Figure S3.** Gating strategy for measurement of in vivo NK cell activation. The gating has been performed in a standardized way, and a typical GPA patient is shown. **a** First, live cells were roughly gated based on forward and sideward scatter (FSC, SSC). Second, Zombie Aqua™ viability dye positive cells were determined as “dead” and remaining cells as “live”. As shown on the bottom, peripheral blood lymphocytes (PBL) were mostly in the live gate, and now re-gated in a conservative, “tight” fashion to exclude monocytes and, as good as possible, potentially apoptotic cells which would be located on the upper left part of the main population. **b** Among PBL, T cells were determined as CD3 + CD19-, B cells as CD3-CD19+ and NK cells as CD3-CD19-CD56+ cells. FMO (“fluorescence minus one”) controls were conducted in all experiments.

## Data Availability

The datasets analyzed during the study are available from the corresponding author on reasonable request.

## Abbreviations

ABA: Abatacept
ADCC: Antibody-dependent cellular cytotoxicity
FCS: Fetal calf serum
FcγRIIIa: Fc-γ-receptor IIIa
GPA: Granulomatosis with polyangiitis
INX: Infliximab
NK cell: Natural killer cell
OBI: Obinutuzumab
PBMC: Peripheral blood mononuclear cell
PBS: Phosphate-buffered saline
RTX: Rituximab
