# Supplementary figures and images for: Activation of natural killer cells by rituximab in granulomatosis with polyangiitis
Source: Arthritis Res Ther. 2019 Dec 11;21:277. doi: 10.1186/s13075-019-2054-0 (PMC6907269; doi:10.1186/s13075-019-2054-0)

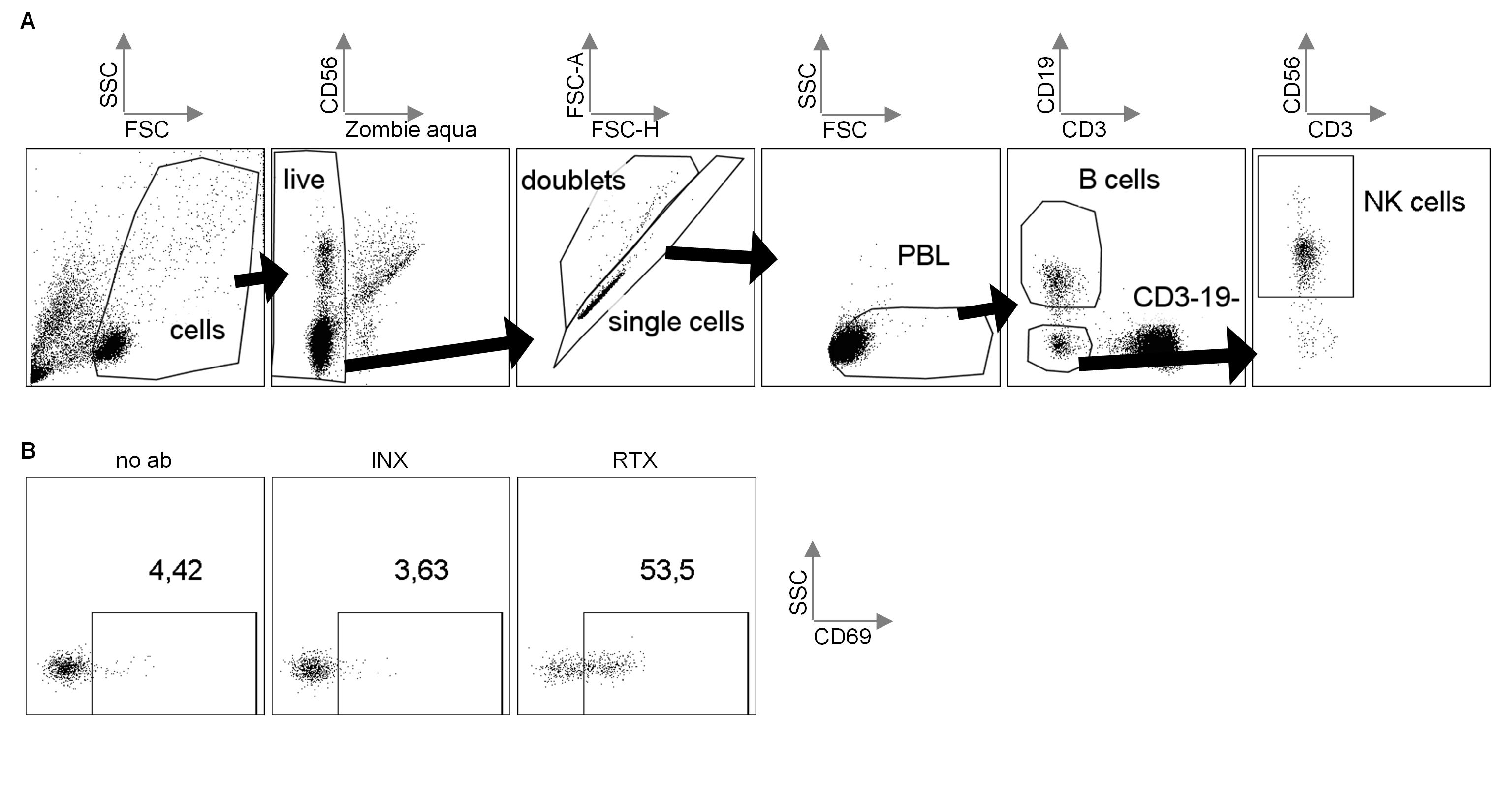

Supplement: Supplementary file 1 — Additional file 1: Figure S1. Gating strategy for in vitro experiments. Gating was performed in a standardized way, and an example GPA patient is shown. a First, live cells were roughly gated based on forward and sideward scatter (FSC, SSC). Second, Zombie Aqua™ viability dye positive cells were determined as “dead” and remaining cells as “live”; for this purpose dot plots showing CD56-Bv421 and Zombie aqua dye were used in order to confirm correct compensation as these fluorochromes were excited by the same laser. After doublet exclusion, peripheral blood lymphocytes (PBL) were gated in a conservative, “tight” fashion to exclude monocytes and, as good as possible, potentially apoptotic cells which would be located on the upper left part of the main population. Among PBL, T cells were determined as CD3 + CD19−, B cells as CD3-CD19+ and NK cells as CD3-CD19-CD56+ cells. b Within the NK cell gate, gates for CD69 positive cells were defined. The same example patient as in a) is shown, after culture with no therapeutic antibody (no ab), with infliximab (INX) or with rituximab (RTX) overnight. Gates for CD107a positive cells and CD16bright cells were defined accordingly. [file 13075_2019_2054_MOESM1_ESM.tif]

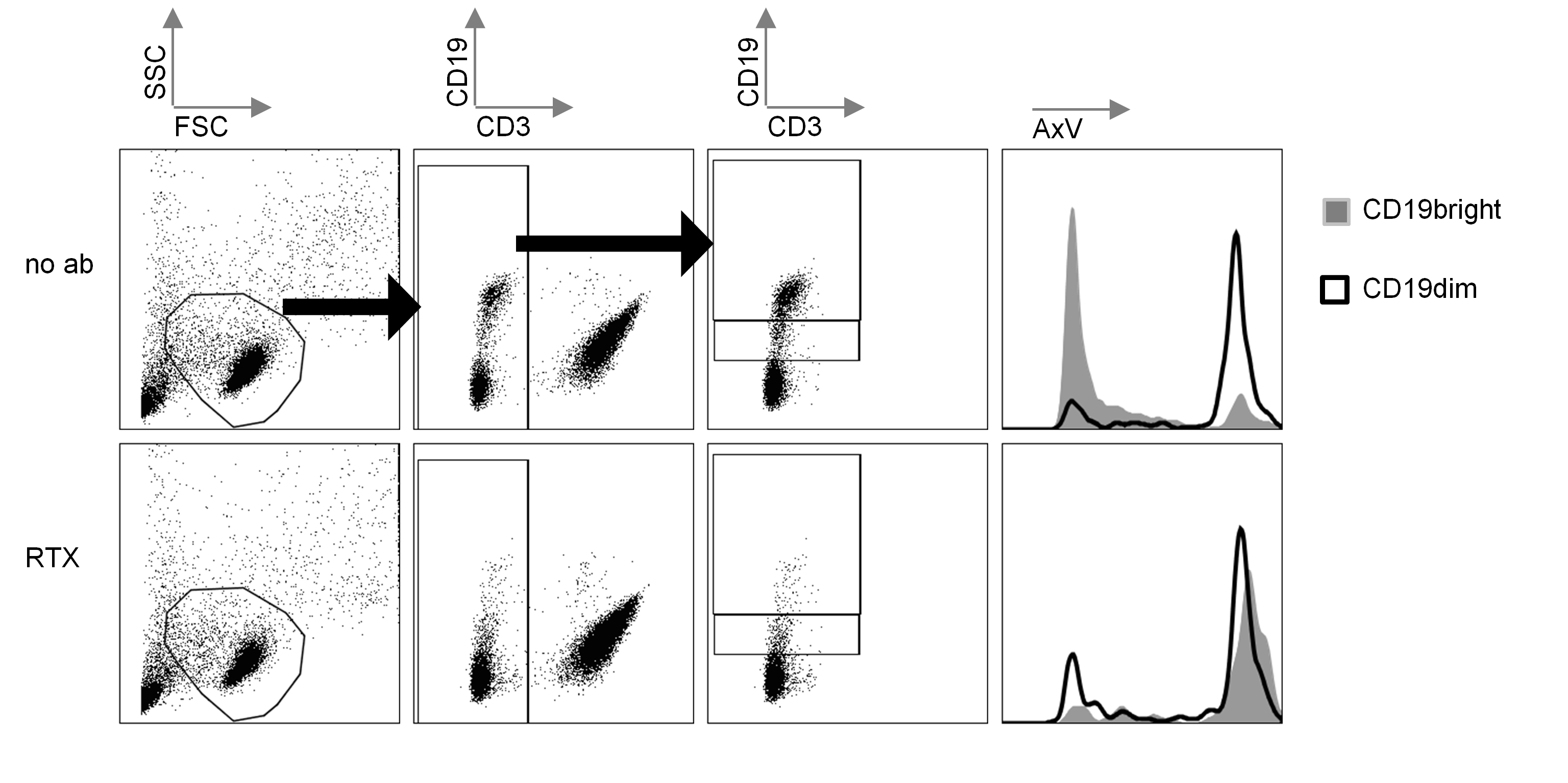

Supplement: Supplementary file 2 — Additional file 2: Figure S2. Loss of CD19 expression was associated with cell death. In order to exclude that reduced numbers of CD19 positive (i.e. CD19 bright) B cells were rather loosing CD19 expression than dying upon incubation with rituximab, PBMCs from healthy donors were incubated without (no ab) or with rituximab (RTX) overnight and subsequently stained with anti-CD3, anti-CD19 and Annexin-V. The gating strategy is shown. The right graphs show overlays of CD3-CD19bright and CD3-CD19dim lymphocytes. Large proportions of CD19dim cells were Annexin-V positive indicating cell death in these cells in both RTX untreated and treated samples. One of three similar experiments is shown. This result was in line with an earlier study [24]. [file 13075_2019_2054_MOESM2_ESM.tif]

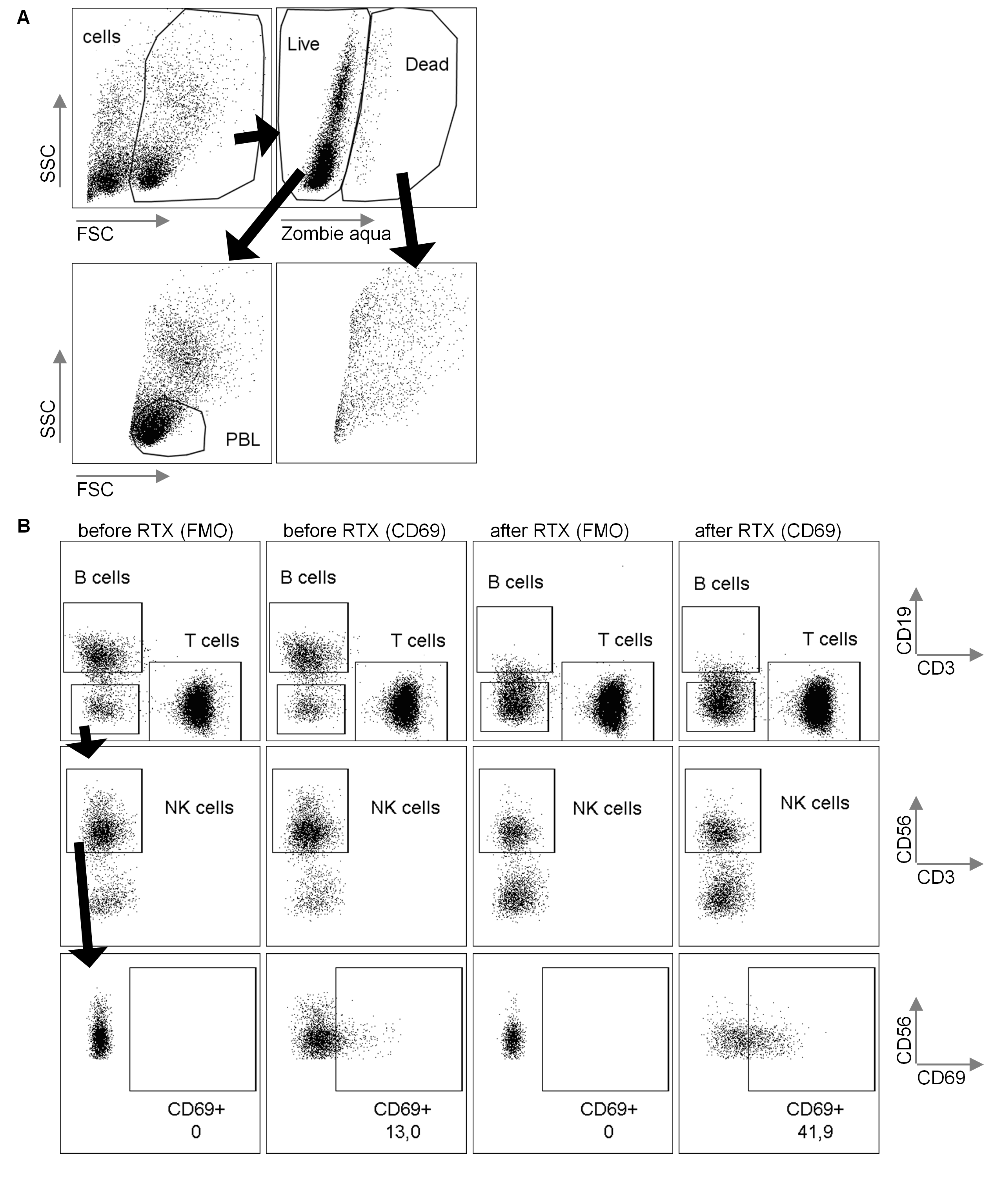

Supplement: Supplementary file 3 — Additional file 3: Figure S3. Gating strategy for measurement of in vivo NK cell activation. The gating has been performed in a standardized way, and a typical GPA patient is shown. a First, live cells were roughly gated based on forward and sideward scatter (FSC, SSC). Second, Zombie Aqua™ viability dye positive cells were determined as “dead” and remaining cells as “live”. As shown on the bottom, peripheral blood lymphocytes (PBL) were mostly in the live gate, and now re-gated in a conservative, “tight” fashion to exclude monocytes and, as good as possible, potentially apoptotic cells which would be located on the upper left part of the main population. b Among PBL, T cells were determined as CD3 + CD19-, B cells as CD3-CD19+ and NK cells as CD3-CD19-CD56+ cells. FMO (“fluorescence minus one”) controls were conducted in all experiments. [file 13075_2019_2054_MOESM3_ESM.tif]
